# Incidence and Clinical Outcomes of Premature Infants: 1‐Year Retrospective Study at the NICU of Gadarif Teaching Hospital

**DOI:** 10.1002/hsr2.73177

**Published:** 2026-09-01

**Authors:** Mohammed Ahmed Abdellah Ahmed, Elshazaly Saeed, Elnazeir Mohamed Ibrahim Mohamedzain, Mohamed Salah Abdulrazeg Abdulrahman, Suleman Sofian Suliman, Ahmed Mohammed Ahmed Abdellah, A. L. Sir Ahmed Addalla Ahmed, Sohaib Mohammed Mokhtar Ahmed

**Affiliations:** ^1^ Department of Pediatric, College of Medicine, King Saud University Celiac Disease Research Chair King Saud University Riyadh Saudi Arabia; ^2^ Faculty of Medicine and Health Sciences University of Gadarif Gadarif Sudan; ^3^ Faculty of Medicine Alzaiem Alazhari University Khartoum Sudan; ^4^ Faculty of Medicine National University Khartoum Sudan; ^5^ Faculty of Medicine University of Khartoum Khartoum Sudan; ^6^ Department of Obstetrics and Gynecology New Halfa Hospital New Halfa Sudan

**Keywords:** NICU, prematurity, Sudan

## Abstract

**Background:**

Prematurity remains a leading cause of neonatal morbidity and mortality worldwide, with the highest burden in low‐resource settings such as sub‐Saharan Africa. Reliable local data are limited in Sudan, particularly in Eastern states. This study aimed to determine the incidence, maternal risk factors, neonatal characteristics, and outcomes of premature infants admitted to the NICU at Gadarif Teaching Hospital.

**Methods:**

A retrospective observational study was conducted from July 2024 to July 2025. Medical records of all neonates admitted to the NICU were reviewed. Preterm birth was defined as delivery before 37 completed weeks of gestation. Maternal, perinatal, and neonatal variables were extracted. Logistic regression models were used to identify predictors of preterm birth and mortality.

**Results:**

Of 604 neonates, 198 (32.8%) were preterm. Mothers of preterm infants were younger (24.3 ± 6.5 vs. 26.4 ± 6.6 years, *p* = 0.002). Elective cesarean delivery and lower parity were associated with prematurity. Pre‐eclampsia was significantly more common among preterm births (4.0% vs. 1.5%, *p* = 0.049). Preterm neonates had markedly lower mean birth weight (1.7 ± 2.4 kg vs. 3.0 ± 1.6 kg, *p* < 0.001) and higher rates of sepsis (71.7%), respiratory distress (62.6%), and jaundice (24.7%). Mortality was significantly higher among preterm infants compared to term (29.4% vs. 7.8%, *p* < 0.001). In multivariable analysis, younger maternal age increased risk of prematurity, while higher gestational age (aOR 0.58, *p* = 0.002) and antenatal care attendance (aOR 0.26, *p* = 0.032) reduced mortality.

**Conclusion:**

Prematurity constituted nearly one‐third of NICU admissions in this cohort and was associated with high mortality. Strengthening antenatal care, delaying early pregnancies, and improving NICU capacity may reduce the burden of preterm related deaths in this setting, although further studies are needed.

## 1 Introduction

Prematurity, defined as birth before 37 completed weeks of gestation, is a major global health challenge and one of the leading causes of neonatal morbidity and mortality. Each year, an estimated 13.4–15 million infants are born preterm, and approximately 1 million die as a result, making prematurity the single largest contributor to under‐five mortality worldwide [1, 2, 3, 4]. The burden is unevenly distributed, with sub‐Saharan Africa and southern Asia together accounting for roughly 65% of all preterm births [1, 3, 4, 5]. Despite this heavy burden, data quality remains a critical limitation in these regions. National surveillance systems often rely on less precise methods of gestational age estimation, such as last menstrual period, which can underestimate the true incidence of preterm birth [1, 5].

In Sudan, and particularly in the eastern states, reliable epidemiological data are scarce. However, evidence from across sub‐Saharan Africa consistently identifies preterm birth as a leading driver of neonatal mortality, especially in rural and underserved areas where health system constraints—limited NICU capacity, shortages of trained staff, and suboptimal antenatal care—delay the recognition and management of high‐risk pregnancies [1, 5, 6]. Reported prevalence rates of preterm birth in sub‐Saharan Africa vary widely (3.4%–49.4%), though most robust estimates cluster between 10% and 12% [1, 5, 6, 7].

Preterm birth is associated with a wide spectrum of adverse outcomes. In the immediate neonatal period, complications such as respiratory distress syndrome, neonatal sepsis, hypothermia, and feeding difficulties are major contributors to NICU admissions and early deaths [5, 7]. Among survivors, long‐term sequelae are common and include neurodevelopmental impairment, cerebral palsy, behavioral and emotional challenges, chronic respiratory disease, and growth failure [5, 8]. These risks are strongly and inversely related to gestational age, with infants born before 32 weeks at highest risk of poor outcomes [1, 3, 8].

The determinants of preterm birth in sub‐Saharan Africa are multifactorial. Key maternal and obstetric risk factors include extremes of maternal age (< 20 or > 35 years), pre‐eclampsia, placental disorders, premature rupture of membranes, chronic maternal disease, previous stillbirth or preterm birth, short interpregnancy interval, multiple gestation, malaria, HIV infection, and inadequate antenatal care (< 4 visits) [5, 6, 9]. Socioeconomic conditions—including rural residence, lower household wealth, and poor access to skilled obstetric services—further amplify risk [5, 6]. Conversely, protective factors such as higher socioeconomic status, marital stability, wanted pregnancy, and adequate antenatal care coverage are associated with lower rates of prematurity [6].

Reducing the burden of prematurity requires context‐specific interventions. Early identification and management of maternal risk factors, strengthening antenatal care coverage, and expanding health system capacity for evidence‐based perinatal and neonatal interventions are critical priorities [3, 5, 7]. High‐quality care, particularly for infants born before 32 weeks, has the potential to avert a substantial proportion of preterm‐related deaths [3]. At the health‐system level, improving data quality, adopting accurate gestational age assessment tools, and strengthening national health information systems are essential to monitor progress and guide policy action [1, 3, 5].

Despite its importance, there remains a lack of high‐quality, up‐to‐date data on the incidence, risk factors, and clinical outcomes of prematurity in Gadarif State. Most available reports are based on small‐scale or outdated hospital data, which limits their utility for guiding local health policy and planning. Understanding the profile and outcomes of preterm infants in this setting is crucial to optimizing resource allocation and designing evidence‐based strategies for neonatal care improvement.

This study aimed to determine the incidence of prematurity among NICU admissions at Gadarif Teaching Hospital, describe the maternal and neonatal characteristics of preterm infants, and identify predictors of mortality in this high‐risk group. By providing local evidence, this work seeks to inform clinical practice and support public health strategies aimed at reducing preterm birth–related morbidity and mortality in Eastern Sudan.

## 2 Methodology

### 2.1 Study Design and Setting

This was a retrospective observational study conducted in accordance with the Strengthening the Reporting of Observational Studies in Epidemiology (STROBE) guidelines. It was conducted at Gadarif Teaching Hospital, the main tertiary referral center in Gadarif State, Eastern Sudan. Gadarif, one of Sudan's 18 states with a population of 2.5 million, borders Ethiopia and sits roughly 400 km from Khartoum. The hospital houses the state's largest public tertiary facility. Its Neonatal Intensive Care Unit (NICU) delivers specialized treatment for critically ill and high‐risk newborns. Staffing includes three pediatricians, eight general practitioners, and thirteen nurses working rotating shifts to maintain round‐the‐clock coverage. Beyond standard neonatal management, the NICU is equipped with nine incubators, nine radiant warmers, and five phototherapy machines, and provides oxygen therapy, umbilical transfusions, nasogastric feeding, IV therapy, urinary catheterization, lumbar puncture, routine lab testing, and continuous positive airway pressure (CPAP) support. The reporting of this study was according to the STROBE guidelines.

### 2.2 Study Population and Participants

The source population comprised all neonates admitted to the NICU during the study period between July 1, 2024 and July 1, 2025. Eligible participants included all live‐born neonates with available records containing gestational age and outcome data. Neonates with missing records on these essential variables were excluded from analyses requiring those data but were retained in the denominator for descriptive statistics when possible. A total of 604 neonates met the inclusion criteria, of whom 198 (32.8%) were preterm and 406 (67.2%) were term. All comparisons between preterm and term infants in this study are restricted to NICU‐admitted infants. Term infants admitted to the NICU represent a high‐risk subgroup (e.g., those with birth asphyxia, meconium aspiration, or infections) and are not representative of all term births in the population. Therefore, findings should not be interpreted as population‐based estimates of risk factors for preterm birth.

### 2.3 Definitions and Outcomes

− Preterm birth was defined as live birth before 37 completed weeks of gestation.
− Gestational age was determined from the clinical records, with most estimates based on maternal last menstrual period (LMP). Early antenatal ultrasound was not routinely available. Where LMP was uncertain or unavailable, clinical assessment (Ballard score) was used. The exact proportion of cases using each method could not be reliably ascertained due to inconsistent documentation.
− Low birth weight (LBW): < 2500 g.
− Maternal risk factors included pre‐eclampsia/eclampsia, gestational diabetes, infections, hypertension, intrauterine growth restriction (IUGR), placental abnormalities, multiple pregnancy, and premature rupture of membranes (PROM).
− Neonatal morbidities included respiratory distress, hypoxic‐ischemic encephalopathy (HIE), meconium aspiration syndrome (MAS), jaundice, hypoglycemia, feeding difficulties, congenital anomalies, and neonatal sepsis which was diagnosed clinically based on the Integrated Management of Childhood Illness (IMCI) criteria, including temperature instability (< 36.5°C or > 37.5°C), lethargy, poor feeding, respiratory distress, and abnormal laboratory findings (C‐reactive protein > 10 mg/L when available). Blood culture was not routinely performed due to resource constraints; therefore, culture‐proven sepsis could not be systematically ascertained.
− Primary outcome: mortality during NICU stay.
− Secondary outcomes: length of stay and discharge status (discharged alive, discharged against medical advice [DAMA], escaped, or transferred).

### 2.4 Data Sources and Measurement

Data were extracted from neonatal medical records using a structured data abstraction form. Variables collected included:

− Maternal characteristics: age, parity, mode of delivery, antenatal care attendance, and pregnancy complications.
− Neonatal characteristics: sex, gestational age, birth weight, immediate crying at birth, and clinical diagnoses.
− Outcomes: length of NICU stay and discharge status.

Data abstraction was conducted by trained research assistants, and ambiguous entries were reviewed by a neonatologist to minimize misclassification bias.

### 2.5 Bias and Study Size

To reduce selection bias, all admissions within the study period were included. The study size was determined by the number of admissions during the study period; no prior sample size calculation was performed as this was a retrospective census of available cases. Missing data were assessed for all study variables. Because the proportion of missing data was low and occurred primarily due to incomplete documentation in the retrospective medical records, a complete‐case analysis was performed for all regression analyses. Observations with missing values for any variable included in a given regression model were excluded only from that analysis. Variables with occasional missing data included antenatal care attendance and some maternal clinical characteristics, whereas gestational age and mortality outcome were complete for all participants included in the analyses.

### 2.6 Quantitative Variables

Gestational age and maternal age were analyzed as continuous variables in regression models. Birth weight was categorized (< 2500 g vs. ≥ 2500 g) in descriptive analyses but retained as continuous when assessing distributions. Length of stay was treated as continuous but presented with mean ± SD.

### 2.7 Statistical Analysis

Data were analyzed using SPSS version 27. Continuous variables were assessed for normality using the Shapiro–Wilk test. Normally distributed data were summarized as mean ± standard deviation (SD) and compared using independent‐samples *t*‐tests or ANOVA. Non‐normally distributed variables were summarized as median with interquartile range (IQR) and compared using Mann–Whitney U tests. Categorical variables were summarized as frequencies and percentages and compared using Pearson's *χ* ^2^ test or Fisher's exact test when appropriate. Incidence of prematurity was calculated as the proportion of preterm admissions among all NICU admissions. Maternal, neonatal, and outcome variables were compared between preterm and term infants.

Two multivariable logistic regression models were developed:

− Model 1 (predictors of preterm birth among NICU‐admitted infants): outcome = preterm versus term. Variables included: maternal age (continuous), parity (continuous), mode of delivery (categorical: elective CS, emergency CS, vaginal delivery), antenatal care attendance (binary), pre‐eclampsia/eclampsia, fetal distress, previous CS, failed trial of labor, multiple pregnancy, placental abnormalities, gestational diabetes, infections, PROM, IUGR, and hypertension.
− Model 2 (predictors of mortality among preterm infants): outcome = death versus survival. Variables included: maternal age (continuous), sex, gestational age (continuous), parity (continuous), antenatal care attendance (binary), cried immediately at birth (binary), HIE, neonatal sepsis, respiratory distress, and neonatal jaundice.

Adjusted odds ratios (aOR) with 95% confidence intervals (CI) were reported. Statistical significance was defined as *p* < 0.05.

## 3 Results

### 3.1 Maternal and Perinatal Characteristics

A total of 604 neonates were included in the study, of which 198 (32.8%) were preterm and 406 (67.2%) were term. The mean maternal age was significantly lower among preterm deliveries compared to term (24.3 ± 6.5 vs. 26.4 ± 6.6 years, *p* = 0.002). Parity did not differ significantly between the two groups (3.1 ± 2.3 vs. 3.2 ± 2.5, *p* = 0.85). Mode of delivery varied significantly, with vaginal delivery accounting for 61.1% of preterm births compared to 47.4% of term births (*p* < 0.001). Antenatal care was less frequent among mothers of preterm neonates, though this difference did not reach statistical significance (84.8% vs. 89.8%, *p* = 0.08. A lower proportion of preterm neonates cried immediately after birth compared to term neonates (62.0% vs. 70.1%, *p *= 0.049) (Table 1).

**Table 1:** Maternal and perinatal characteristics of NICU‐admitted infants by gestational status.

Variable	Term (*n* = 406)	Preterm (*n* = 198)	*p* value
Age of mother, mean (SD)	26.4 (6.6)	24.3 (6.5)	0.002
Parity, mean (SD)	3.2 (2.5)	3.1 (2.3)	0.85
Mode of delivery			< 0.001
Elective C‐section	68 (17.0%)	7 (3.5%)	
Emergency C‐section	143 (35.7%)	70 (35.4%)	
Normal vaginal delivery	190 (47.4%)	121 (61.1%)	
Antenatal care (R‐ANC)			0.08
Yes	336 (89.8%)	156 (84.8%)	
No	38 (10.2%)	28 (15.2%)	
Sex of infant			0.17
Male	260 (65.8%)	117 (60.0%)	
Female	135 (34.2%)	78 (40.0%)	
Cried immediately			0.049
Yes	272 (70.1%)	119 (62.0%)	
No	116 (29.9%)	73 (38.0%)	

### 3.2 Maternal Risk Factors

Pre‐eclampsia/eclampsia was more common among preterm deliveries (4.0%) compared to term (1.5%, *p* = 0.049). Placental abnormalities were also more frequent among preterm births (3.5% vs. 1.2%), although this difference did not reach statistical significance (*p* = 0.057). Gestational diabetes occurred more frequently among term births (6.9% vs. 1.0%, *p *= 0.002). Other maternal risk factors, including multiple pregnancy, infections, PROM, gestational diabetes, and hypertension, did not differ significantly between groups (Table 2).

**Table 2:** Maternal risk factors among NICU‐admitted infants by gestational status.

Variable	Term (*n* = 406)	Preterm (*n* = 198)	*p* value
Pre‐eclampsia/eclampsia	6 (1.5%)	8 (4.0%)	0.049
Multiple pregnancy	20 (4.9%)	16 (8.1%)	0.12
Placental abnormalities	5 (1.2%)	7 (3.5%)	0.057
Gestational diabetes	28 (6.9%)	2 (1.0%)	0.002
Infections	69 (17.0%)	41 (20.7%)	0.27
PROM	63 (15.5%)	31 (15.7%)	0.97
Hypertension	9 (2.2%)	6 (3.0%)	0.55

### 3.3 Neonatal Characteristics

Preterm neonates had a mean gestational age of 31.8 ± 3.6 weeks. Their mean birth weight was markedly lower compared with term neonates (1.7 ± 1.1 kg vs. 3.0 ± 1.6 kg, *p* < 0.001). The prevalence of low birth weight (< 2500 g) was significantly higher among preterm infants (93.9%) compared to term (25.1%, *p* < 0.001). Hypoxic‐ischemic encephalopathy (HIE) was less frequent in preterm neonates (6.6%) compared to term (24.4%, *p* < 0.001). Meconium aspiration syndrome (MAS) was also significantly less common in preterm neonates (2.5% vs. 23.4%, *p* < 0.001). Conversely, neonatal sepsis (71.7% vs. 58.4%, *p* = 0.001), respiratory distress (62.6% vs. 44.1%, *p* < 0.001), and neonatal jaundice (24.7% vs. 11.3%, *p *< 0.001) were more frequently observed in preterm infants (Table 3).

**Table 3:** Neonatal characteristics of NICU‐admitted infants by gestational status.

Variable	Term (*n* = 406)	Preterm (*n* = 198)	*p* value
Birth weight, mean (SD), kg	3.0 (1.6)	1.7 (1.1)	< 0.001
Low birth weight (< 2500 g)	102 (25.1%)	186 (93.9%)	< 0.001
Hypoxic‐ischemic encephalopathy	99 (24.4%)	13 (6.6%)	< 0.001
Meconium aspiration syndrome	95 (23.4%)	5 (2.5%)	< 0.001
Neonatal sepsis	237 (58.4%)	142 (71.7%)	0.001
Respiratory distress	179 (44.1%)	124 (62.6%)	< 0.001
Neonatal jaundice	46 (11.3%)	49 (24.7%)	< 0.001

### 3.4 Outcomes

The mean length of stay in the NICU was significantly longer for preterm neonates compared with term neonates (6.0 ± 7.2 vs. 3.2 ± 4.3 days, *p* < 0.001). Mortality was higher among preterm infants (29.4% vs. 7.8%, *p *< 0.001). Among preterm neonates, 58.4% were discharged alive, 9.6% were discharged against medical advice, 2.5% escaped, and none were transferred to another facility. Among term neonates, 82.2% were discharged alive, 6.0% were discharged against medical advice, 2.0% escaped, and 2.0% were transferred (Table 4).

**Table 4:** Outcomes of NICU admission by gestational status.

Outcome	Term (*n* = 406)	Preterm (*n* = 198)	*p* value
Length of stay, mean (SD), days	3.2 (4.3)	6.0 (7.2)	< 0.001
Death	31 (7.8%)	58 (29.4%)	< 0.001
Discharged alive	328 (82.2%)	115 (58.4%)	
Discharged against advice	24 (6.0%)	19 (9.6%)	
Escaped	8 (2.0%)	5 (2.5%)	
Transferred	8 (2.0%)	0 (0.0%)	

### 3.5 Multivariable Analysis

In the regression model predicting preterm birth, maternal age, mode of delivery, and parity were significant predictors. Maternal age was associated with higher odds of preterm delivery (aOR 1.08, 95% CI 1.03–1.14, *p* = 0.002). Compared to elective cesarean delivery, both emergency cesarean (aOR 0.06, 95% CI 0.007–0.47, *p* = 0.008) and vaginal delivery (aOR 0.03, 95% CI 0.004–0.29, *p* = 0.002) were associated with reduced odds of preterm birth. Increasing parity was also associated with lower odds of preterm delivery (aOR 0.84, 95% CI 0.73–0.96, *p *= 0.012) (Table 5).

**Table 5:** Multivariable logistic regression predicting preterm birth among NICU‐admitted infants (vs. term NICU‐admitted infants).

Predictor	aOR (95% CI)	*p* value
Maternal age (per year)	1.08 (1.03–1.14)	0.002
Mode of delivery		
Emergency C‐section vs. elective	0.06 (0.007–0.47)	0.008
Normal vaginal delivery vs. elective	0.03 (0.004–0.29)	0.002
Parity (per unit increase)	0.84 (0.73–0.96)	0.012
Antenatal care (yes vs. no)	1.72 (0.79–3.72)	0.17
Pre‐eclampsia/eclampsia (yes vs. no)	0.14 (0.01–1.61)	0.12
Fetal distress (yes vs. no)	2.56 (0.44–14.8)	0.30
Previous C‐section (yes vs. no)	0.73 (0.27–2.01)	0.55
Failed trial of labor (yes vs. no)	1.00 (0.26–3.84)	0.99
Multiple pregnancy (yes vs. no)	0.54 (0.14–2.05)	0.37
Placental abnormalities (yes vs. no)	0.31 (0.05–1.93)	0.21
Gestational diabetes (yes vs. no)	2.41 (0.27–21.9)	0.44
Infections (yes vs. no)	0.69 (0.37–1.27)	0.23
PROM (yes vs. no)	0.98 (0.51–1.89)	0.95
IUGR (yes vs. no)	0.40 (0.06–2.53)	0.33
Hypertension (yes vs. no)	1.35 (0.20–9.13)	0.76

In the regression model predicting mortality among preterm infants, gestational age and antenatal care were significant predictors. Each additional week of gestational age was associated with reduced odds of mortality (aOR 0.58, 95% CI 0.41–0.81, *p* = 0.002). Receipt of antenatal care was similarly associated with lower mortality risk (aOR 0.06, 95% CI 0.007–0.57, *p *= 0.014). Other maternal and neonatal variables were not statistically significant in the adjusted model (Table 6).

**Table 6:** Multivariable logistic regression predicting mortality among preterm infants.

Predictor	aOR (95% CI)	*p* value
Maternal age (per year)	0.96 (0.89–1.04)	0.34
Sex (male vs. female)	0.49 (0.19–1.25)	0.13
Gestational age (per week)	0.58 (0.41–0.81)	0.002*
Parity (per unit increase)	1.13 (0.91–1.41)	0.26
Antenatal care (yes vs. no)	0.26 (0.07–0.89)	0.032*
Cried immediately (yes vs. no)	1.05 (0.38–2.89)	0.92
HIE (yes vs. no)	0.90 (0.12–7.05)	0.92
Neonatal sepsis (yes vs. no)	1.63 (0.44–6.04)	0.47
Respiratory distress (yes vs. no)	1.07 (0.34–3.33)	0.91
Neonatal jaundice (yes vs. no)	1.40 (0.49–4.01)	0.53

## 4 Discussion

This study provides valuable evidence on the incidence, risk factors, neonatal characteristics, and outcomes of premature infants admitted to the NICU at Gadarif Teaching Hospital, Eastern Sudan. The findings demonstrate a substantial burden of prematurity among NICU‐admitted infants in this setting, which may reflect broader maternal and neonatal health challenges. These observations suggest potential priorities for intervention that warrant further investigation.

The incidence of prematurity among NICU admissions in this study was 32.8%, which is substantially higher than the global estimate of 10%–12% reported for sub‐Saharan Africa and worldwide [10]. This elevated proportion may reflect referral bias, as the NICU serves as a tertiary center where high‐risk deliveries are concentrated. Nevertheless, it underscores the heavy burden of prematurity in Eastern Sudan, aligning with previous reports from low‐resource African settings where prematurity contributes disproportionately to neonatal morbidity and mortality [11]. Such high incidence calls for strengthened antenatal care coverage and preventive strategies targeting modifiable maternal risk factors.

Younger maternal age was significantly associated with preterm delivery in this cohort, while higher parity reduced the odds of prematurity. These findings are consistent with evidence from large‐scale analyses across sub‐Saharan Africa, which identify maternal age under 20 years and primiparity as important risk factors for preterm birth [6]. Biologically, young maternal age is linked to incomplete pelvic growth, hormonal immaturity, and inadequate prenatal care utilization, all of which contribute to early delivery. Higher parity may confer protective effects through physiologic adaptation to prior pregnancies. Public health strategies that delay early pregnancies through education and reproductive health programs may therefore reduce preterm birth rates in Sudan.

Elective cesarean section was strongly associated with prematurity compared to vaginal delivery and emergency cesarean section, but the small number of elective cesarean deliveries in the preterm group (*n* = 7) resulted in very wide confidence intervals. Therefore, this association should be interpreted cautiously as hypothesis‐generating rather than definitive. A larger prospective study is needed to clarify the relationship between elective cesarean delivery and prematurity in this setting. However, this mirrors global patterns where elective cesarean delivery—often performed for maternal or fetal indications such as pre‐eclampsia, malpresentation, or prior cesarean—increases the risk of iatrogenic prematurity [12]. In low‐resource contexts, poor access to timely ultrasound dating may further exacerbate the problem by leading to inaccurate gestational age estimation and unnecessary early delivery [13]. This finding highlights the need to strengthen pregnancy dating methods in Sudan, particularly through wider access to early ultrasound.

Pre‐eclampsia/eclampsia was significantly more frequent among preterm deliveries, confirming its role as a leading cause of medically indicated preterm birth [14]. Hypertensive disorders contribute to placental insufficiency and intrauterine growth restriction, necessitating early delivery to save maternal and neonatal lives. Interestingly, gestational diabetes was more common in term births, diverging from global trends where it is often linked to increased prematurity risk [15]. This discrepancy may reflect underdiagnosis of gestational diabetes in resource‐limited Sudanese settings, with only severe cases being detected, or differences in population risk profiles.

Premature infants were significantly more likely to experience sepsis, respiratory distress, and jaundice, while term infants had higher rates of hypoxic‐ischemic encephalopathy (HIE) and meconium aspiration syndrome (MAS). These results are consistent with established pathophysiology: immature lungs and immune systems predispose preterm infants to respiratory distress and infection [16, 17], whereas term neonates are more likely to suffer intrapartum complications such as HIE and MAS due to prolonged labor and meconium passage [18]. These findings reinforce the need for improved infection prevention protocols, timely administration of surfactant therapy, and enhanced neonatal resuscitation training in Sudanese NICUs.

In this cohort, preterm infants had significantly longer NICU stays and higher mortality compared to term infants (29.4% vs. 7.8%). This survival gap mirrors low‐ and middle‐income countries findings where mortality inversely correlates with gestational age and birth weight [19]. The clinical implication is that even small gains in prolonging gestation and improving intrauterine growth could meaningfully improve survival.

Multivariable analysis revealed that higher gestational age and antenatal care attendance significantly reduced the odds of mortality among preterm infants. Each additional week of gestation conferred a 42% reduction in mortality odds, in line with regional and global survival data [20, 21]. Antenatal care likely improves survival by enabling earlier detection of complications, administration of antenatal corticosteroids, and timely referral for delivery. However, ANC coverage in this study was suboptimal, with 15.2% of mothers of preterm infants reporting no ANC visits. Expanding access to quality antenatal services in rural Sudan should therefore be a policy priority to reduce preventable preterm‐related deaths.

The similar prevalence of PROM between preterm and term NICU‐admitted infants (15.7% vs. 15.5%) is notable. This may reflect selection bias: term infants admitted to the NICU represent a high‐risk subgroup, and PROM in term infants that leads to NICU admission likely represents complicated PROM (e.g., associated with chorioamnionitis, prolonged rupture > 18 h, or fetal distress). In contrast, uncomplicated term PROM may not result in NICU admission. This underscores the importance of interpreting all comparisons as restricted to NICU‐admitted infants only.

## 5 Limitations

This study has several limitations that should be considered when interpreting the findings. First, its retrospective design relied on medical record review, which may have introduced information bias due to incomplete or inconsistent documentation. Gestational age estimation was primarily based on maternal last menstrual period, which is less accurate than early ultrasound and could have led to misclassification of preterm status. Second, the study was conducted in a single tertiary hospital, which may limit the generalizability of the results to the broader population of Eastern Sudan, particularly rural communities with limited access to NICU services. Third, some maternal risk factors such as socioeconomic status, nutritional status, and lifestyle factors were not captured in the records, restricting the ability to fully assess their contribution to prematurity. An additional limitation is that the medical records did not consistently document the method used for gestational age estimation (LMP vs. ultrasound vs. clinical examination). Most estimates were based on LMP, which is less accurate than early ultrasound. This may have led to misclassification of preterm status, and the lack of standardized documentation precludes a precise breakdown of methods used. Lastly, the study did not follow neonates beyond hospital discharge, so long‐term outcomes such as neurodevelopmental impairment could not be evaluated. Despite these limitations, the study provides important baseline evidence for prematurity in this under‐researched region and highlights key areas for future prospective research.

Furthermore, this study included only neonates admitted to the NICU, representing a selected population of high‐risk infants requiring specialized care. Consequently, the reported incidence of prematurity, associated risk factors, and clinical outcomes should not be interpreted as representative of all newborns or the general neonatal population in Eastern Sudan. The findings should therefore be interpreted within the context of NICU‐admitted infants and extrapolated with caution.

## 6 Recommendations

Based on the findings of this study, several measures may be considered based on these findings to reduce the burden of prematurity and improve neonatal outcomes in Eastern Sudan. First, strengthening antenatal care coverage and quality is essential, with emphasis on early booking, routine risk assessment, and timely management of maternal complications such as pre‐eclampsia. Expanding access to accurate gestational age assessment through ultrasound would help prevent iatrogenic prematurity associated with elective cesarean sections. At the community level, interventions that delay early pregnancies, promote birth spacing, and improve maternal nutrition should be prioritized. Within NICUs, scaling up infection prevention protocols, respiratory support technologies, and staff training in neonatal resuscitation can reduce preventable deaths. These recommendations should be integrated into broader health system strategies to improve maternal and neonatal health in resource‐limited settings such as Gadarif.

## 7 Conclusion

This study demonstrates that prematurity represents a major clinical and public health challenge in Eastern Sudan, accounting for nearly one‐third of NICU admissions and carrying a disproportionately high risk of morbidity and mortality. Key maternal factors—including younger age, low parity, and hypertensive disorders—were significantly associated with preterm delivery, while neonatal outcomes highlighted the vulnerability of premature infants to sepsis, respiratory distress, and prolonged hospitalization. Gestational age and antenatal care emerged as strong protective factors against mortality, underscoring the critical role of preventive maternal health services. Addressing these challenges may benefit from a two‐pronged approach: strengthening maternal care to reduce the incidence of prematurity and improving neonatal intensive care capacity to enhance survival. With targeted interventions, it is possible to significantly reduce preterm‐related mortality and improve newborn survival in Sudan.

## Author Contributions

**Mohammed Ahmed Abdellah Ahmed:** conceptualization, methodology, validation, funding acquisition, writing – original draft. **Elshazaly Saeed:** conceptualization, writing – review and editing, funding acquisition. **Elnazeir Mohamed Ibrahim Mohamedzain:** investigation, writing – original draft. **Mohamed Salah Abdulrazeg Abdulrahman:** writing – review and editing, writing – original draft. **Suleman Sofian Suliman:** writing – original draft, data curation, investigation. **Ahmed Mohammed Ahmed Abdellah:** writing – original draft, data curation, resources. **A. L. Sir Ahmed Addalla Ahmed:** writing – original draft, writing – review and editing. **Sohaib Mohammed Mokhtar Ahmed:** conceptualization, methodology, formal analysis, data curation, supervision, writing – review and editing.

## Ethics Statement

The study protocol was submitted to and approved by the Ethics Committee of the Faculty of Medicine, University of Gadarif. All extracted data were anonymized at the point of data entry; unique identifiers were removed, and the electronic dataset was stored on a password‐protected computer accessible only to the study team. No individual patient identifiers are reported in the manuscript.

## Conflicts of Interest

The authors declare no conflicts of interest.

## Transparency Statement

The corresponding author, Sohaib Mohammed Mokhtar Ahmed, affirms that this manuscript is an honest, accurate, and transparent account of the study being reported; that no important aspects of the study have been omitted; and that any discrepancies from the study as planned have been explained.

## Data Availability

Data used in this study are available upon reasonable request from the corresponding author.
